# Application of CRISPR/Cas9 in Alzheimer’s Disease

**DOI:** 10.3389/fnins.2021.803894

**Published:** 2021-12-21

**Authors:** Likui Lu, Xi Yu, Yongle Cai, Miao Sun, Hao Yang

**Affiliations:** Institute for Fetology, The First Affiliated Hospital of Soochow University, Suzhou, China

**Keywords:** gene therapy, CRISPR/Cas9, cognitive function, Alzheimer’s disease, delivery system

## Abstract

Alzheimer’s disease (AD) is a progressive and irreversible neurodegenerative disorder clinically characterized by cognitive impairment, abnormal behavior, and social deficits, which is intimately linked with excessive β-amyloid (Aβ) protein deposition along with many other misfolded proteins, neurofibrillary tangles formed by hyperphosphorylated tau protein aggregates, and mitochondrial damage in neurons, leading to neuron loss. Currently, research on the pathological mechanism of AD has been elucidated for decades, still no effective treatment for this complex disease was developed, and the existing therapeutic strategies are extremely erratic, thereby leading to irreversible and progressive cognitive decline in AD patients. Due to gradually mental dyscapacitating of AD patients, AD not only brings serious physical and psychological suffering to patients themselves, but also imposes huge economic burdens on family and society. Accordingly, it is very imperative to recapitulate the progress of gene editing-based precision medicine in the emerging fields. In this review, we will mainly focus on the application of CRISPR/Cas9 technique in the fields of AD research and gene therapy, and summarize the application of CRISPR/Cas9 in the aspects of AD model construction, screening of pathogenic genes, and target therapy. Finally, the development of delivery systems, which is a major challenge that hinders the clinical application of CRISPR/Cas9 technology will also be discussed.

## Introduction

Alzheimer’s disease (AD) is a progressive and irreversible neurodegenerative disorder that is characterized by cognitive dysfunction, abnormal behavior, social deficits, and an eventual inability to perform daily tasks. It is predicted that by 2050, the number of Alzheimer’s patients aged 65 years and older in the U.S. may reach 13.8 million (No authors listed, 2020). In China, more than 15.07 million people aged 60 years or older have dementia, and approximately 9.83 million of them suffer from AD (Jia et al., 2020). Accordingly, so many AD patients will bring major public health challenges. And to make matters worse, the pathogenesis of AD is currently still unclear. Until now, β-amyloid protein deposition and tau protein hyperphosphorylation are widely regarded as key contributions to the neurobiological mechanisms underlying the pathogenesis of AD (Mila-Aloma et al., 2020; Zetterberg and Bendlin, 2021). Besides, other age-related, protective, and disease-promoting factors probably interact with the core mechanisms of AD and may be involved in the onset of AD. For instance, emerging extensive research has shown that some disregarded partners such as vascular dysfunction (Sweeney et al., 2019), oxidative stress (Tonnies and Trushina, 2017), proteinopathy (Sharma and Kim, 2021), metal ions (Huang et al., 2015; Liu et al., 2019b), neuroinflammation (Calsolaro and Edison, 2016), mitochondrial dysfunction (Mantzavinos and Alexiou, 2017; Perez Ortiz and Swerdlow, 2019), and microbiota-gut-brain axis (Cryan et al., 2019) are also closely associated with the AD progress.

Due to the complexity of the pathogenesis, the clinical manifestations of AD vary greatly between individuals. In addition to cognitive impairment, it also involves many neuropsychiatric symptoms including apathy, aggression, depression, anxiety, sleep disorders, hallucination, and irritability. These symptoms require active treatment when they cause serious adverse effects, however, the presently clinical drugs available merely delay the progression of AD even though some have certain efficacy, but the ultimate misfortune outcome is inevitable. Meanwhile, the irreversibility of the disease process also brought great challenges to developing more effective therapeutic interventions in AD. At present, the global researchers in the field are committed to seeking potential biomarkers for early diagnosis of AD and has sought to expand the mechanism of AD progress, and attempts to reverse the progress of AD through efficient gene editing therapy.

**FIGURE 1:**
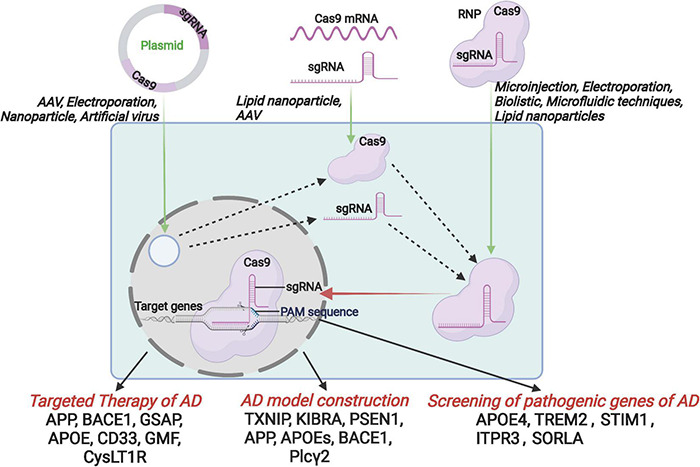
Schematic diagram of the introduction of CRISPR/Cas9 components into host cells by the format of CRISPR/Cas9 plasmid, mRNA and RNP, and further application for the targeted therapy, AD model construction and the screening of pathogenic genes.

Recently, CRISPR/Cas9 (Clustered Regularly Interspaced Short Palindromic Repeats, CRISPR associated nuclease, CRISPR/Cas) technology is developing rapidly and showing a great potential in the field of basic research and disease therapeutics. Also, the gene editing technology was evaluated as a promising approach in AD research and treatment (Giau et al., 2018; Hanafy et al., 2020). This review first will introduce the development and advantages of CRISPR/Cas9 technology, and then focus on the application of CRISPR/Cas9 in the aspects of AD model construction, screening of pathogenic genes, and target therapy. Finally, we have also summarized the development of delivery systems, which is a major challenge that hinders the clinical application of CRISPR/Cas9 technology (Figure 1).

## CRISPR/Cas9 Gene-Editing Technology

### The Composition and Principle of CRISPR/Cas9 System

The CRISPR/Cas9 system is an adaptive bacterial and archaeal defense mechanism that recognizes and disables invading bacteriophages or other foreign nucleic acids (Giau et al., 2018). In general, the gene-editing systems are divided into two classes. The class 1 system contains types I, III and IV, and the class 2 system contains types II, V, and VI (Gupta et al., 2019). Type II CRISPR/Cas9 is the most routinely used for CRISPR gene-editing system and usually refer to CRISPR. The CRISPR/Cas9 system is composed of the Cas9 protein and the sgRNA (single guide RNA, sgRNA). In this system, the sgRNA guides the system to the target, and the Cas9 can cleave the double strands of DNA (Liu et al., 2017). The sgRNA is essential for precise gene editing as its 5′-terminal 20-nucleotide sequence interacts with a target sequence of host DNA *via* Watson and Crick base pairing rules, while its 3′ duplex structure allows binding to Cas9 proteins (Jinek et al., 2012). The sgRNA is composed of the trans-activating crRNA (tracrRNA) and crRNA. The crRNA contains a 20-nt protospacer element and an additional sequence that is complementary to the tracrRNA. The tracrRNA hybridizes to the crRNA and binds the Cas9 protein, forming the Cas9-sgRNA complex to create double-stranded breaks (DSBs) at target sites in the genome (Cong et al., 2013; Yang et al., 2013; Dow et al., 2015). The Cas9 protein has six domains including REC I, REC II, Bridge Helix, PAM-interacting, HNH and RuvC. The protospacer adjacent motif (PAM) is a short sequence of nucleotides directly adjacent to the target DNA sequence. The Cas9 nucleases from different bacterial species recognize different PAM sequences for seeking targets, with SpCas9 using “NGG” PAM as a binding target while SaCas9 employing “NNGRRT” PAM (Lino et al., 2018; Xie et al., 2018). The HNH and RuvC domains are nuclease domains that cut single-stranded DNA. The RuvC domain cleaves non-complementary DNA strands, while the HNH domain cleaves complementary DNA strands (Jinek et al., 2012). This system presumably functions *via* the high nuclease activity of CRISPR/Cas9 that induces highly efficient, targeted double-stranded breaks (DSBs). Following a DSB, there are multiple fates for the broken chromosomal DNA. Primary DSB repair mechanisms include non-homologous end joining (NHEJ) or homology directed repair (HDR) pathways (Kaulich et al., 2015). NHEJ involves direct ligation of the two broken chromosomal DNA strands to one another and is the main cellular DSB repair mechanism. This process is error-prone owing to random insertions or deletions (indels) of nucleotides to assist in ligation, which can cause gene disruption *via* frameshift or nonsense mutations (Lieber, 2010). The HDR pathway performs DNA repair through homologous recombination and rarely mismatches in replicating DNA.

### The Development of CRISPR/Cas9 System and Its Advantages Over Other Gene-Editing Technologies

The first CRISPR was cloned from *E. coli* by accident in 1987. Ishino et al. (1987) noted the presence of a 29-nucleotide repeat sequence in Escherichia coli, which was interrupted by unrelated, non-repetitive short sequences (spacers). These regularly spaced motifs were clustered next to the *lap* gene, which encodes an aminopeptidase in Escherichia coli K12. Two years later, a second array was found in the same genome, and hybridization assays suggested the presence of similar sequences in very close relatives (Shigella and Salmonella species) (Nakata et al., 1989). In 1991, interspaced direct repeats (DR) were identified in strains of an evolutionarily distant group of bacteria, the Mycobacterium tuberculosis complex (MTBC) (Hermans et al., 1991). The DR-intervening sequences, known as spacers, were found to differ among isolates, and hence were harnessed for strain typing (Jeffreys et al., 1991; Groenen et al., 1993). Later the similar CRISPR sequences were also cloned from other bacteria and Archaea (Hermans et al., 1991; Mojica et al., 1993; Jansen et al., 2002). However, at the first almost one decade, the scientists did not know the function of these special repeat sequences and just thought they are a special sequence among different species and used them as strain typing.

In 2000, the presence of similar repeats was reported in most prokaryotes and was then named “CRISPR” by Ruud Jansen (Mojica et al., 2000; Jansen et al., 2002). The significance of the spacers being derived from foreign genetic elements was established in 2005 (Mojica et al., 2005). In 2012, two independent laboratories reported that *in vitro* reconstructed CRISPR/Cas systems had biological functions and were capable of cleaving an individual DNA sequence (Gasiunas et al., 2012; Jinek et al., 2012). This provides foundation for using CRISPR/Cas as a genome editing tool. By January 2013, three independent US teams led by Luciano Marraffini (Jiang et al., 2013), Feng Zhang (Cong et al., 2013) and George Church (Mali et al., 2013) succeed in editing the bacterial and mammalian genomes using Cas9.

In recent years, based on the discovery of a variety of high-efficiency nucleases, gene editing technology has been rapidly developed and widely used. The artificial endonuclease-mediated genome editing technology developed most rapidly (Gupta and Shukla, 2017), mainly including 4 types: meganuclease technology (meganuclease), zinc finger nuclease technology (zinc finger nuclease, ZFN), transcription activator-like effector nuclease technology (transcription activator-like effector nuclease, TALEN) and clustered regularly interspaced short palindromic repeats (CRISPR-associated proteins, Cas) system. Different from ZFNs, TALENs and Meganucleases identifying and linking to the DNA to create a double-strand break by the proteins, the binding domain of the CRISPR/Cas9 deriving from RNA implies that the system is more straightforward, efficient and convenient (Khadempar et al., 2019). In this review, we summarized the advantages and limitations of these gene-editing methods (see Table 1). With the development of this technology, CRISPR/Cas9 gene editing system has been extensively applied to gene engineering such as genes knocking-in of mammalian cells (Banan, 2020), human diseases modeling (Torres-Ruiz and Rodriguez-Perales, 2017) and gene therapy, etc. In recent years, the CRISPR/Cas9 technology has improved dramatically over the past decade, and the efficiency of CRISPR/Cas9 system can be greatly increased by lipid-encapsulated gold nanoparticles as a multifunctional vehicle enabling a more effective therapy for tumor (Wang et al., 2018a). By means of the application of artificial virus, CRISPR/Cas9 system has many advantages such as minimum side effects, high efficiency and security over other superinfect and lipofectamine methods (Li et al., 2017). In conclusion, CRISPR/Cas9 technology is increasingly mature and it has the potential to become an effective tool for gene editing in clinical therapy.

**TABLE 1:** Comparison of different gene-editing methods.

	CRISPR/Cas9	ZFNs	TALENs	Meganucleases	RNAi
Nuclease	Cas9	FOKI	FOKI	I-SceI	Dicer and Argonaute proteins
Recognition mechanism	PAM sequences and the complementary sgRNA site-DNA	Zinc finger proteins-DNA	RVDs- DNA	Protein-DNA	RNA
Cycle	Short	Long	Long	Long	Long
Cytotoxicity	Low	High	Low	Low	Variable to high
Versatility	High	High	High	Limited	Not mentioned
Cost	Low	High	High	Low	Low
Delivery	Easily	Limited	Limited	Limited	Limited
RNA editing	Yes	No	No	No	Yes
Specificity	High	High	High	High	High
Stability	High	High	High	High	Low
Application case	AD target therapy	CCR5 gene mutagenesis resisting HIV	Cystic Fibrosis-Gene Therapy	Embed artificial gene networks	Antiviral therapy

## CRISPR/Cas9 in Alzheimer’s Disease

In recent years, due to the short experimental period and relatively low consumption of CRISPR/Cas9 technology, CRISPR/Cas9 is currently widely used in the AD field including construction of AD model, screening pathogenic genes, and target therapy.

### Alzheimer’s Disease Model Construction

Cell models are widely used in studies of various neurological diseases including AD because this does not involve ethical concerns, and the experimental period is relatively short, and the cost is low as well. In the past decades, numerous *in vitro* cell models of AD were established. To date, cell lines commonly used in AD research include human neuroblastoma cells SH-SY5Y and SK-N-SH, mouse hippocampal neuron cell lines HT22 and glial cells BV2, and mouse glioblastoma cells N2a. The emergence of CRISPR/Cas9 gene editing technology can facilitate more efficiently developing AD cell models. Wang et al. (2019) reported that down-regulation of Thioredoxin-interacting protein (Txnip) level in HT22 cells *via* CRISPR/Cas9 system can effectively attenuate amyloid-β-induced protein cysteine oxidative modification. The findings indicated that Txnip may be a therapeutic target for the treatment of AD. Meanwhile, Song et al. (2019) also found that knockdown of KIBRA (KIdney and BRAin expressed protein) in HT22 cells suppressed its growth and caused apoptosis while treatment with Aβ_1–42_ oligomers. In addition, Sun et al. (2017) revealed that knock-out of Presenilin 1 (PSEN1) genes in N2a cells by using CRISPR/Cas9 system can eliminate the background of endogenous γ-secretase, and found that exogenous addition of recombinant protein derived from PSEN1 mutations can decrease the production of Aβ42 and Aβ40. However, owing to the differences in genome and physiology characteristics between these cell lines and genuine neural cells, the constructed AD model using these cells still cannot precisely elucidate the molecular mechanism responsible for AD occurrence. Therefore, patient-derived induced pluripotent stem cells (iPSCs) have received more and more attention due to their unique physiological profiles. In 2016, Paquet et al. (2016) introduced mutations of amyloid precursor protein (APP) and PSEN1 which cause AD, into human iPSCs. Subsequently, the higher Aβ levels were detected in the converted neurons from the mutant homozygous and heterozygous human iPSCs compared with isogenic controls, implying the AD-associated mutations could be modeled in human neural cells by CRISPR/Cas9 technology.

Apart from building AD cell models, CRISPR/Cas9 technology can also be used to build AD animal models. In 2020, Serneels et al. (2020) created a novel model named mouse and rat Apphu/hu by using CRISPR/Cas9 strategy to generate a humanized Aβ sequence (G676R, F681Y, and R684H) in APP gene of mouse and rat. And by inserting the three amino acids in the rodent Aβ sequence, the level of Aβ increased more than three times compared to original WT strain. Apolipoprotein E (APOE) has different variants including APOE2, APOE3, APOE3r, and APOE4. Komor et al. (2016) transfer APOE3r into APOE4 in mouse astrocytes by converting C in codon 158 to T *via* CRISPR/Cas9 system, which implicates the point mutations being modified by CRISPR/Cas9 method. Until 2019, Cas9 nanocomplexes with sgRNAs targeting tyrosine hydroxylase (Th) and β-secretase 1 (BACE1) genes were delivered into mouse primary neurons to assess the efficiency of Cas9 nanocomplexes by Park et al. (2019). Excitingly, almost none of off-target was found by using a variety of survey assays, Sanger sequencing, and deep sequencing. Concomitantly, they injected Cas9-BACE1 nanocomplexes into the hippocampal of 6-month-old AD mouse. Four weeks later, a remarkable decrease of BACE1 expression and β-cleavage products of APP was found in the hippocampal of the AD mice (Park et al., 2019). Moreover, Takalo et al. (2020), designed a Plcγ2-P522R knock-in mouse model by using CRISPR/Cas9 gene editing strategy and assessed the protective effects of Plcγ2-P522R variant. Their results revealed that the microglial functions were enhanced in Plcγ2-P522R knock-in mice, implying that the gene editing strategy is likely to be a new treatment approach for AD (Takalo et al., 2020).

Non-human primates are one of the most suitable models for human diseases owing to their genetic, physiological and pathological similarities with human beings. In 2014, several researchers from China succeeded in achieving two gene modifications (Rag1 and Ppar-γ) of cynomolgus monkeys by CRISPR/Cas9 system at one step. Inspiringly, no off-target mutations were found. Soon afterward, the gene modification of both ear punch tissues and placenta are also conducted, demonstrating the success of global gene modification (Niu et al., 2014). Next year, the Cas9-mediated gene modifications were substantiated in monkey germline, implying the possibility that the artificial modifications are transmitted to their offspring (Chen et al., 2015). For more thorough gene knockout, the injection of various sgRNAs is more effective than single type of high concentration sgRNAs. The thorough gene knock-out by CRISPR/Cas9 system is conducive to reduce the test times of non-human primates and guarantee the accuracy, which is strongly supported by ethics (Zuo et al., 2017). Although no AD primate models were designed *via* CRISPR/Cas9 technology, however, to further study on the pathogenesis of AD and more efficiently build AD primate models, it is necessary to construct a primate AD model by CRISPR/Cas9 strategy.

### Screening of Pathogenic Genes for Sporadic Alzheimer’s Disease

Alzheimer’s disease is classified into sporadic (sporadic AD, SAD) and familial (familiar AD, FAD). APP, PSEN1, and PSEN2 are the main pathogenic genes caused FAD, and the mutation in each gene could contribute to the onset of FAD. However, more than 95% of AD cases are sporadic (Zhang et al., 2020). The pathogenesis is very complicated, involving genetic factors (such as APOE), and is associated with age, environmental factors and many diseases including diabetes (Baglietto-Vargas et al., 2016) and vascular diseases (Zetterberg and Mattsson, 2014). Therefore, the screening of SAD pathogenic genes is crucial for understanding the molecular pathogenesis, early diagnosis, risk prediction, and treatment of AD.

The development of high-throughput sequencing and CRISPR/Cas9 has provided great help for the screening of SAD pathogenic genes. The APOE gene is currently recognized as a susceptibility gene for SAD. Wadhwani et al. (2019) used CRISPR/Cas9 system to correct the E4 allele into E3/E3 genotype in iPSCs from 2 AD patients, and found that E3 neurons are more resistant to ionomycin-induced cytotoxicity. However, E4 cells exhibited an increase in tau phosphorylation and contributed to the calcium dysregulation and ultimately cell death (Wadhwani et al., 2019). This finding intensifies our understanding of the relationship between APOE4 and the pathogenesis of SAD. In addition, Kolay and Diamond (2020) reported that knock-out of the 22 genes (derived from GWAS) associated with AD *via* CRISPR/Cas9 in HEK293T cells had no effect on the tau metabolism. A recent study conducted by the lab of the University of California Irvine reveals the crucial roles of TREM2 in the pathogenesis of AD (McQuade et al., 2020). They found that knock-out of TREM2 in iPSCs *via* CRISPR system, the survival of microglia, the clearance of APOE and SDF-1α/CXCR4-mediated chemotaxis was seriously impacted, which ultimately caused the impairment response to beta-amyloid plaques. As for the function of AD-associated TREM2 variant, Cheng-Hathaway et al. (2018) generate a mouse model of AD encoding the one copy R47H variant in TREM2 *via* CRISPR/Cas9 method, and found the heterozygous AD mouse showed reduced expression of TREM2 in cells and decreased myeloid cell responses to amyloid deposition, indicating the relationship between TREM2 R47H variant and increased risk of AD. Beyond that, researchers recently also found that p.S1038C variant may strengthen the risk of AD when a homozygous mutation (rs377155188, C > G, p.S1038C) was introduced in TTC3 gene *via* CRISPR/Cas9 system (Laverde-Paz et al., 2021). Furthermore, in order to investigate the function of STIM1 gene in neurodegeneration, Pascual-Caro et al. (2019) establish a STIM1 knock-out SH-SY5Y neuroblastoma cells model by CRISPR/Cas9 system, and demonstrated that once knock-out of STIM1 gene, mitochondrial function abnormality and calcium homeostasis imbalance rapidly occurred, ultimately leading to cell death. Notably, the expression of STIM1 is significantly reduced in the brain of patients with AD, and STIM1-deficient SH-SY5Y cells further substantiated the relationship between STIM1 gene and the onset of AD *in vitro*. In 2020, Pascual-Caro et al. (2020) found a remarkable reduction of ITPR3 following the knock-out of STIM1 gene, which caused the mitochondrial function abnormality. This further confirmed the potential role of STIM1-ITPR3 axis in the pathogenesis of AD (Pascual-Caro et al., 2020). Similarly, Knupp et al. (2020) found that the depletion of SORLA in hiPSCs by using CRISPR/Cas9 can cause early endosome enlargement in hiPSCs-derived neurons rather than hiPSC microglia, and inhibition of BACE is not able to rescue the endosome enlargement phenotype in hiPSC neurons, indicating the independent function of SORLA in AD pathogenesis. More convenient is a design of Duan et al. (2021) which is imaging based arrayed CRISPR to investigate the genes related to AD features. This will also provide a platform to explore the AD biology and an opportunity for drug discovery (Duan et al., 2021).

### Targeted Therapy

AD is the most common neurodegenerative disease in the elderly. Cortical neuritic plaques and the neurofibrillary tangles are two neuropathological hallmarks of AD (Glenner and Wong, 1984; Masters et al., 1985). The neurofibrillary tangles are composed of hyperphosphorylated tau protein. The neuritic plaques are generally the result of the excessive cleavage of the APP by BACE1. Herein, we will mainly discuss the gene therapy strategies for AD (see Table 2), which focus on the regulation of Aβ expression by using CRISPR/Cas9 technology.

**TABLE 2:** Targeted therapy of AD *via* CRISPR/Cas9 system.

Target genes	Main results	Model system	Delivery system	References
Amyloid precursor protein (APP)	APP↓ Aβ↓	Tg2576 mice as mutant models of APP familiar form of Alzheimer	Adeno-associated viral (AAV)-1 vectors	Gyorgy et al., 2018
3′-UTR APP	APP↓ Aβ↓	C57BL/6 mice	px330 plasmid	Nagata et al., 2018
beta-secretase 1 (Bace1)	Bace1↓ Aβ↓ Memory impairment↓	5XFAD as Alzheimer mouse model and wild-type mice	Micelle	Park et al., 2019
γ-Secretase activating protein (GSAP)	GSAP↓ γ-Secretase activity↓ Aβ↓	HEK-APP cell lines	Plasmid	Wong et al., 2019
APOE	Turning APOE4 to APOE3↑ Hyper-phosphorylation Tau protein↓ Deposition of amyloid ↓	Induced pluripotent stem cells (iPSCs)	Electroporation with three episomal plasmids	Wadhwani et al., 2019
CD33	hCD33m+ hCD33M- Aβ1–42 phagocytosis in microglia↑	U937 cells	Not mentioned	Bhattacherjee et al., 2021
Glia maturation factor (GMF)	GMF↓ p38 MAPK ↓	BV2 microglial cell line	AAVpro	Raikwar et al., 2019
CysLT1R	CysLT1R^–/–^ hippocampal synaptic plasticity↑ amyloidogenesis↓ neuroinflammation in the hippocampus↓	APP/PS1 mice	Not mentioned	Chen et al., 2021

#### CRISPR/Cas9 Targets APP Gene Mutations

The mutation in the APP gene causes dominantly inherited AD as a result of increased β-secretase cleavage of the amyloid-β (Aβ) precursor protein. The KM670/671NL APP mutation, indigenous to Sweden (APPsw for the mutation and APPsw for the mutant allele), results in an increase in enzymatic cleavage by β-secretase, and thereby increased Aβ protein levels. Gyorgy et al. (2018) reported that the expression of Aβ protein decreases when APP alleles were knocked out using CRISPR/Cas9 technology. Therefore, the CRISPR/Cas9 system may provide gene therapy strategies for AD patients with APP mutations. Moreover, Nagata et al. (2018) identified possible protective deletion mutations in the 3′-UTR of the APP gene in mice. They found a drastic reduction of Aβ accumulation when ∼700-bp of the 891-bp APP 3′-UTR in the mouse model zygotes was deleted using CRISPR/Cas9 technology. Interestingly, the A673T mutation is the reason for an Icelandic population that did not show symptoms of AD at an advanced age. This mutation can reduce β-secretase cleavage by 40% (Jonsson et al., 2012). Accordingly, Guyon et al. (2021) hypothesized that the insertion of this mutation in patients’ neurons could be an effective and sustainable method of slowing down or even hindering the progression of AD. To this end, they introduced a new mutation using CRISPR/Cas9-based strategy to modify the APP gene by converting the alanine codon to a threonine in HEK293T cells and SH-SY5Y cells (containing the APP gene with deaminated cytosine1 and cytosine2 positions). The accumulation of Aβ peptide have further reduced owing to a successful introduction of the A673T mutation in 53% of HEK293T cells alongside a new mutation (E674K). Likewise, in Sun’s laboratory (Sun et al., 2019), they also selectively edited endogenous APP at the extreme C-terminus using a CRISPR/Cas9-based strategy in cell and animal models, and reciprocally manipulated the amyloid pathway. Therefore, the Aβ production have been reduced by attenuating APP-β-cleavage, while elevating neuroprotective APP-α-cleavage.

#### CRISPR/Cas9 Targets Key Enzymes of Aβ Protein

##### CRISPR/cas9 Nanocomplex Targets BACE1

Aβ protein is formed by sequential modification of APP through BACE1 and γ-secretase. Thereby targeting BACE1 is a potential therapeutic strategy for the treatment of AD. Park et al. (2019) reported that the expression of BACE1 has been successfully reduced by employing Cas9 nanocomplexes, which was prepared by adding amphiphilic R7L10 peptide to Cas9-sgRNA, in two mouse models of AD.

##### CRISPR/cas9 Targets γ-Secretase Protease

Another target of gene therapy in AD is a large intramembrane protein complex known as γ-secretase protease which is regulated by γ-secretase activating protein (GSAP). There is evidence to suggest that reduction in GSAP expression decreases Aβ levels significantly (He et al., 2010; Ghosh and Surolia, 2011). Based on the claims, Wong et al. (2019) also knocked out GSAP with CRISPR-Cas9 technology in HEK293 cells that stably express APP (HEK-APP), leading to a remarkable reduction in Aβ secretion and γ-secretase activity.

Given that γ-secretase is regulated by the expression of the GSAP, and PSEN1 and PSEN2 are the key components of the γ secretase complex. Therefore, the mutations of PSEN1 would cause AD and be associated with most familial AD (Raux et al., 2005; Wu et al., 2011; Chavez-Gutierrez et al., 2012; Wang et al., 2018b). Most of these mutations impair amyloid metabolism, resulting in an elevation in Aβ42/40 ratio and Aβ42 levels, and/or reduced production of Aβ40 (Hung and Livesey, 2018). Also, PSEN1 gene mutations have been proven to be linked with the majority of early-onset familial AD. The statement was confirmed by Fang et al. (2006) who found a novel V97L missense mutation at codon 97 (Val97Leu) of the PSEN1 gene in a Chinese familial AD pedigree. To verify whether this mutation is pathologically functional, they established a mutation type of SH-SY5Y cell line *via* CRISPR/Cas9 system and detected Aβ production. Strikingly, the level of Aβ42 was significantly elevated both intracellularly and extracellularly in mutation type SH-SY5Y cells at 48 h when compared to wild type cells. This indicates that the mutation of PSEN1 is a potential factor of AD pathogenesis. In addition, Ortiz-Virumbrales et al. (2017) reported that cell lines harboring the PSEN2 N141I mutation displayed an increase in the Aβ42/40 in iPSC-derived basal forebrain cholinergic neurons (BFCNs). More importantly, increased Aβ42/40 was normalized following CRISPR/Cas-mediated correction of the N141I mutation in PSEN2.

#### CRISPR/Cas9 Targets Editing of APOE Genotype

The APOE4 isoform is the strongest genetic risk factor for SAD (Huang et al., 2017). As we know, APOE is mainly expressed by astrocytes in the central nervous system. However, APOE expression in neurons will imply occurrence of these events including the age-related cognitive impairment, neurological injury, and neurodegeneration. A study regarding therapeutic target for APOE4 by Wadhwani et al. (2019) showed that when the E4 allele was corrected to E3/E3 genotype in iPSCs from two patients with AD *via* CRISPR/Cas9 method, E3 neurons were less susceptible to ionomycin-induced cytotoxicity, and exhibited a decrease in tau phosphorylation. Furthermore, Lin et al. (2018) identified APOE4 function by using hiPSC and CRISPR/Cas9 technology, their results showed that APOE4 had an impact on the Aβ metabolism in a cell-type-specific manner by different ways. More exciting results showed that isogenic conversion of APOE4 to APOE3 can attenuate multiple AD-related pathologies (Lin et al., 2018). These findings further revealed that APOE4 is a promising target for the treatment of AD.

#### CRISPR/Cas9 Targets Proinflammatory Molecules

##### CRISPR/cas9 Targets CD33

Human genetic association studies indicate that immune response is also the major pathway of AD etiology. The importance of chronic neuroinflammation in AD, has been demonstrated by accumulating evidence. CD33, an immunomodulatory receptor, is expressed at high levels on neutrophils and low levels on microglia and has divergent roles in regulating phagocytosis responsible for AD pathology (Griciuc et al., 2013; Estus et al., 2019). Recent research by Bhattacherjee et al. (2019) showed that the genetic ablation of mCD33 enhanced microglia phagocytosis to increase Aβ clearance *via* CRISPR/Cas9 technology in U937 (highly expressing hCD33M) and attenuated the pathological phenotype of AD. Furthermore, their team disrupted CD33 gene using CRISPR/Cas9 and complemented with different variants of hCD33, leading to the alleviation of Aβ pathology and neurodegeneration. On the contrary to hCD33M that represses phagocytosis, hCD33m is a variant of hCD33 and improve Aβ phagocytosis (Bhattacherjee et al., 2021). These results provided strong support that AD-protective CD33 allele will facilitate AD therapeutics targeting these receptors.

##### CRISPR/cas9 Targets Glia Maturation Factor

Glia maturation factor (GMF), a newly discovered pro-inflammatory molecule, is predominantly expressed in the reactive glial cells surrounding the amyloid plaques and highly expressed in various AD brain regions (Ahmed et al., 2017). Overexpression of GMF usually leads to neuronal cell death by activating the p38 MAPK signaling pathway and oxidative toxicity. Raikwar et al. (2019) successfully reduced GMF expression in BV2 cells *via* CRISPR/Cas9 method, resulting in inhibition of pp38 MAPK to regulate GMF-induced proinflammation in microglia.

##### CRISPR/cas9 Targets cysLT1R

Cysteinyl leukotrienes (Cys-LTs) are a group of the inflammatory lipid molecules and initiate inflammatory signaling cascades by two major G-protein coupled receptors (CysLT1R and CysLT2R). In the recent years, mounting evidence shows that CysLT1R is intimately associated with the occurrence and development of AD, and can mediate inflammatory response *via* the NF-κB pathway (Yu et al., 2005; Wang et al., 2011). Reportedly, the high level of Aβ1–42 significantly causes the elevation of CysLT1R expression. The CysLT1R antagonist can remarkably suppress the overexpression of CysLT1R-mediated inflammatory response *via* the NF-κB pathway. Chen et al. (2021) also demonstrated that the deletion of CysLT1R *via* CRISPR/Cas9 system reduces amyloid pathology and alleviates neuroinflammation in APP/PS1 mice. As a result, Aβ1-42-induced cognitive and hippocampal synaptic impairments in APP/PS1 mice were ameliorated. In summary, this study will provide an insight into elucidation of the mechanism underlying CysLT1R-mediated AD pathology.

### Delivery System of CRISPR/Cas9 in Alzheimer’s Disease

For therapeutic application, the CRISPR components must be delivered to mammalian cells to allow gene alteration in the host cell. Although considerable advances have been made in the *in vivo* administration of CRISPR/Cas9, the biocompatibility, safety and tissue specificity remain challenging. Relying on the genetic modification desired, different components of CRISPR/Cas9 are delivered. Amongst the simplest implementation, the Cas9/sgRNA pair is sufficient to disrupt genes (such as knock-out), however, the delivery of an additional piece of DNA is required for advanced bio-functions such as gene repair or insertion (knock-in). The CRISPR/Cas9 components can be delivered into cells in various forms: viral, mRNA, plasmid, and protein-based approaches. In the subsequent sections, we will further discuss the strengths and limitations regarding these potentially clinically translatable approaches, as well as their own separate potential for clinical applications.

#### Viral Delivery Methods

Viral methods are the most broadly used approaches for delivery of CRISPR/Cas9. At the present, many virus delivery systems have been utilized to deliver CRISPR/Cas9 including adeno-associated viruses (AAV), Adenoviral Vectors (AdV) and Lentiviral Vectors (LV) (Xu et al., 2019).

##### Adeno-Associated Viruses

Adeno-associated virus (AAV), one of the smallest viruses, is originally discovered as a contaminant of purified adenovirus preparation, and its replication is dependent on adenovirus due to a failure to encode a polymerase, thereby they have been called AAV (Rose et al., 1966). Along with numerous classes of viral vectors, AAVs have been widely used for CRISPR genome editing, the specific reasons are multiple. Firstly, after entering the cell, most of AAV is free from the host cell genome. Once integrated into the host genome, the provirus will be able to stably and continuously express for 1–2 years, which is relatively beneficial for the treatment of diseases. Secondly, AAV can widely infect different tissues due to the distinct capsids. Weinmann et al. (2020) has identified a myotropic AAV-9 variant by massively parallel *in vivo* evaluation of barcoded capsid variants, which exhibits superior efficiency and specificity in the musculature including skeletal muscle, heart and diaphragm following peripheral delivery. Thirdly, AAV can tolerate pH and temperature changes, and constantly keep its stable activity (Rayaprolu et al., 2013). To date, the only limitation seems to be the formulated concentration for delivery (Wright, 2008).

The robust stability of these vectors provides ample options for different administration routes and specialized delivery strategies. Researchers have delivered gene-editing components to mdx mice (a model of DMD) after birth by adeno-associated virus-9 (AAV9), finally leading to modification of the mutant dystrophin gene, and achieved success in treating DMD *in vivo* (Long et al., 2016). In addition, an open-label clinical trial proved that bilateral stereotactic administration of AAV2-NGF (CERE-110) to the nucleus basalis of Meynert could produce long-term, biologically active NGF expression, and AAV2-NGF was safe and well-tolerated for 2 years. Hence, AAV2-NGF may become an effective target for the treatment of AD (Rafii et al., 2014). CERE-120, also known as an adeno-associated virus type-2 (AAV2) vector encoding NTN, is currently being developed as a potential therapy for neurodegenerative disease. It was reported that when monkeys received bilateral injections of CERE-120 across a different range of doses, caused a dose-related increase in NTN protein expression within the striatum and substantia nigra (SN) pars compacta including nigrostriatal tyrosine hydroxylase (Th). Besides, the phosphorylated signal-regulated kinase responsible for common neurotrophic signaling event was activated (Herzog et al., 2008). Furthermore, Marks et al. (2016) provide evidence for the long-term safety of CERE-120 gene transfer.

One of the major limitations of AAVs is the small genome-packaging capacity of ∼4.7 kb. Since many diseases are caused by genes whose coding sequence exceeds this capacity, packaging into a single AAV capsid is currently not feasible for these genes. To overcome the size limit of AAV vector, one strategy is to split large transgenes into two or three parts, generating dual or triple AAV vectors (Akil, 2020); the other strategy is to use a smaller Cas9 variants that allow for the packaging of genes encoding both Cas9 and sgRNA into a single vector (Ran et al., 2015). Nevertheless, no method is currently perfect, and there are still many limitations to the application of AAV. As for dual or triple AAV vector strategy, the accumulation of mRNA (Xu et al., 2004) and low transduction levels (Maddalena et al., 2018) have become new obstacles. In addition, a smaller Cas9 variant from streptococcus aureus Cas9 (saCas9) could cause the body to produce humoral and cell-mediated adaptive immunity, again hindering its therapeutic application (Charlesworth et al., 2019).

##### Adenoviral Vectors

Adenoviridae is a medium-sized virus, about 90–100 nm large, is an icosahedral DNA virus without a mantle, and has a nucleocapsid. The genetic material of adenovirus is linear double-stranded DNA with a total length of about 30,000–42,000 bp (Wold and Toth, 2013). There are at least 57 serotypes of human Ads (Adenoviruses), Ad1-Ad57, that form seven “species” A–G (Wold and Toth, 2013). Ads mainly infect a variety of vertebrates, including humans, leading to lifelong immunity. In addition, there are no drugs approved specifically to treat Ad infections. Although Ad is dangerous, AdV was found to have many advantages. For instance, AdV can grow into high titer stable stocks and persist in their expression extrachromosomally rather than by integrating into the host genome (Wold and Toth, 2013). Furthermore, gene delivery vectors that do not rely on host cell genome integration could avoid insertion mutation and position effect variegation (Athanasopoulos et al., 2017).

Nonetheless, AdV also have certain shortcomings, for instance, a well-known highly immunogenicity. Early clinical trials for gene correction using AdV did not yield several successes in clinical therapy, and one trial resulted in a tragic fatality (Raper et al., 2003). In order to avoid this serious situation from happening again, Capasso et al. (2014) further demonstrated that making chemical modifications in AdV facilitated overcoming some of the early challenges regarding liver targeting and host immunity. Nevertheless, things are two-sided, as for cancer gene therapy, the strongly immune responses are beneficial for therapeutic outcomes. Therefore, AdV have recently been used in cancer treatment as oncolytic viruses. Many clinical trials use AdV to target a number of different cancers, such as prostate, ovarian, bladder, and refractory solid tumors (Burke et al., 2012; Kim et al., 2013; Freytag et al., 2014; Hemminki et al., 2015).

##### Lentiviral Vectors

Retroviruses are single-stranded RNA viruses whose genome is reverse transcribed into double-stranded DNA and integrated into the infected cell genome. Genomic integration leads to stable maintenance and potentially sustained expression. These features make them particularly appreciated when the stable, long-term expression was sought. The basic genes required for retroviral and lentiviral survival and function are the gag, pol, and env genes (Escors and Breckpot, 2010). In addition, the lentivirus genus of retroviruses includes a variety array of accessory genes (Delenda, 2004). The lentiviruses that infect various mammals have been transformed into lentiviral vectors, but the most used are those based on HIV-1 (Naldini et al., 1996). With the continuous development of lentiviral vectors, the safety of the third-generation lentiviral vectors is greatly improved (Milone and O’Doherty, 2018).

Retrovirus shows a very important role in clinical gene therapy. For instance, direct clinical benefit with chimeric antigen receptor T (CAR-T) cells is a promising novel therapy for many malignancies, such as leukemia and lymphoma (Schuster et al., 2019; Sterner et al., 2019). CAR-T cells are produced by *ex vivo* transduction of T cells with lentiviral vectors. Schuster et al. (2019) have found an exciting result that high rates of durable responses were produced with the use of tisagenlecleucel in relapsed or refractory diffuse large B-cell lymphoma in adults. However, because CD19 is a pan-B cell marker, one side effect is normal B-cell depletion. Nevertheless, CAR-T cell therapy is still an attractive alternative to treating or preventing leukemia and lymphoma. Following CAR-T cell therapy, another sensational study recently demonstrated the infinite possibilities of lentiviral vectors. The researchers used a lentiviral-mediated gene therapy program-LentiGlobinBB305 to treat patients with β-thalassemia and achieved significant curative effects. Meanwhile, they showed that gene therapy with lentiviral vectors is safe. Therefore, this new gene therapy is likely to rescue or reduce long-term blood transfusion for patients with transfusion-dependent beta thalassemia (TDT) (Thompson et al., 2018).

#### Plasmid-Based Approaches

Delivery of DNA encoding the Cas9 protein is an attractive way to introduce the CRISPR/Cas9 machinery into the cells. This method has advantages as below: firstly, gene synthesis is relatively simple; secondly, the synthesized gene does not need integrating into the host genome after being transferred into the host cell through a plasmid, and can be continuously expressed. Furthermore, the organ specific delivery of the CRISPR/Cas9 system is very important for the further application. One potential advantage of plasmid-based delivery is that tissue or cell-specific targeting can also be integrated into the plasmid itself.

In 2020, Lu et al. reported a first-in-human phase I clinical trial of CRISPR/Cas9 PD-1-edited T cells in patients with advanced non-small-cell lung cancer (Lu et al., 2020). In this clinical trial, the plasmids expressing two sgRNAs and Cas9 edited hPD-1 plasmids were co-transfected into T cells by electroporation. After infusion of edited T cells, the treatment-related adverse events were grade 1/2, indicating the safety and feasibility of therapeutic application of CRISPR/Cas9 in non-small-cell lung cancer.

Despite promising reports of the utility of CRISPR/Cas9 for *in vivo* gene editing, a principal problem in implementing process is how to deliver high molecular weight DNA to cells. At the present, Jo et al. (2020) designed a PLGA nanoparticle fluorescently labeled with the fluorophore 6, 13-bis (triisopropylsilylethynyl) pentacene (TIPS pentacene) to delivered Cas9 into primary bone marrow derived macrophages. Inspiringly, the expression of Cas9 protein was initially found after 24 h, and TIPS fluorescence was detected in most cells (Jo et al., 2020). In addition, Wang et al. (2018a) reported a strategy to deliver Cas9-sgPlk-1 plasmids (CPs) which is condensed on TAT peptide-modified Au nanoparticles (AuNPs/CP, ACP) *via* electrostatic interactions, following lipid-encapsulated and laser-controlled. This method for CRISPR/Cas9 delivery is highly efficient (Wang et al., 2018a). These studies indicate that the method for nanoparticles coating plasmids is likely to effective in the *in vivo* therapeutic application of CRISPR/Cas9. Besides, researchers have constructed a multifunctional nucleus-targeting “core-shell” artificial virus (RRPHC) to delivery CRISPR/Cas9 plasmid. The system can induce a higher targeted gene disruption than traditional transfection reagent (such as Lipofectamine 3000). More excitingly, the artificial virus can effectively target ovarian cancer *via* dual-receptor-mediated endocytosis (Li et al., 2017). Therefore, this will provide an ideal idea for efficient delivery and targeting of CRISPR/Cas9.

#### RNA-Based Delivery

Delivery of Cas9-encoded mRNA is another commonly used approach for the introduction of CRISPR machinery into the cells. Unlike gene-based delivery methods, mRNA-based strategies are transient in functioning, and circumventing the risks associated with integration into the host genome (Nelles et al., 2016). mRNA-based methods also benefit from more rapid effect since mRNA is transcribed in a matter of minutes (Zetsche et al., 2015). However, the obvious challenges regarding the maintenance of RNA stability and delivery of each component of CRISPR/Cas9 individually remains.

Recently, Beijing National Laboratory for Molecular Science has reported an CRISPR/Cas9 delivery nanocarrier called BAMEA-O16B that can efficiently deliver Cas9 mRNA and sgRNA into target cells. After intravenous injection of BAMEA-O16B/Cas9 mRNA/sgRNA nanoparticle, the lipid nanoparticle can efficiently accumulate in the liver, leading to a significant decrease of the proprotein convertase subtilisin/kexin type 9 in mouse serum (Liu et al., 2019a). Library of engineered LNPs (lipid nanoparticles) containing barcoded mRNA (b-mRNA) has been designed by Guimaraes et al. (2019) which enables direct barcoding and subsequent quantification of a functional mRNA, can accelerate the *in vivo* screening and design of LNPs for mRNA therapeutic applications such as CRISPR/Cas9 gene editing. These further broaden the therapeutic prospects of mRNA-based CRISPR/Cas9 technology. In addition, a previous study reported a combinatorial delivery method. In this study, researchers used lipid nanomaterials to deliver Cas9 mRNA, and used adeno-associated virus to deliver sgRNA and a repair template. Finally, treatment alleviated the disease symptoms in the mouse model of the human hereditary tyrosinemia (Yin et al., 2016). This result further shows the joint multiple delivery methods may be an effective strategy for the clinical application of CRISPR/Cas9 technology in the future, which can remedy the shortcomings of a single delivery method.

To improve genome-editing efficiency by increasing sgRNA stability, further studies reported the benefits of synthetic modifications to the sgRNA (Yin et al., 2017). This represents an effective method to overcome the stability issues associated with RNA-based CRISPR/Cas9 delivery.

#### Protein-Based CRISPR/Cas9 Strategies

In CRISPR/Cas9 gene editing technology, ribonucleoprotein (RNP) consisting of Cas9 protein and sgRNA is a powerful method for genome editing, which has various advantages including fast and safe, lower off-targeting, and higher editing efficiency. In addition, the Cas9 RNP system can be applied to various model organisms and cell types, such as stem cells (Wang et al., 2021), immune cells (Schumann et al., 2015), primary cells (Seki and Rutz, 2018), etc.

At the present, there are many options including physical approaches (such as microinjection, electroporation, biolistic, and microfluidic techniques) and synthetic carriers (lipid nanoparticles) can be used for the delivery Cas9 RNP system. Nevertheless, microinjection has strict requirements, cumbersome process and high cost. Hence the method needs to be further improved. More encouragingly, a recent work conducted by Chen et al. (2016) showed a simple and economic electroporation-based strategy to delivery Cas9 RNP system. Compared to microinjection, CRISPR RNP Electroporation of Zygotes (CRISPR-EZ) can efficiently increase the embryo viability (Chen et al., 2016). As for lipid nanoparticles to delivery Cas9 RNP, the lab of Hokkaido University has reported a lipid nanoparticle (LNP)-based CRISPR/Cas ribonucleoprotein (RNP) combined microfluidic techniques to delivery Cas9 and Cpf1 RNPs. The delivery technique following optimized formula, can efficiently suppress both HBV DNA and covalently closed circular DNA (cccDNA) in HBV-infected human liver cells (Suzuki et al., 2021). These progressively findings have made significant contributions to the development of the CRISPR/Cas9 delivery system and its therapeutic and clinical applications.

The above-mentioned delivery strategies are not actually independent of each other. Each delivery strategy has its own merits and limitations (see Table 3). Lattanzi et al. (2019) compared the different delivery systems-based CRISPR/Cas9 into HSPCs (hematopoietic stem and progenitor cells). Results showed that plasmid-based method has high editing efficiency but was associated with significant cell toxicity. RNA-mediated way has similar cell toxicity to plasmid-based method, and has less editing efficiency. Although LVs-based delivery system has minimal cell toxicity, the genome-editing efficiency is low. By contrast, RNP-based delivery of CRISPR/Cas9 exhibits a good balance between cytotoxicity and editing efficiency. Above all, it is often necessary to jointly apply these delivery methods to achieve desired result.

**TABLE 3:** Generalized comparison of different CRISPR/Cas9 delivery format.

Delivery format	Delivery vehicle	Advantages	Editing efficiency	Immunogenicity	Limitations	References
Plasmid	Electroporation; nanoparticle; AAV; artificial virus	Gene synthesis is simple; no need to integrate into the genome; tissue or cell-specific targeting	Moderate	Moderate	Low capacity	Li et al., 2017; Thompson et al., 2018; Wang et al., 2018a; Lu et al., 2020
mRNA	Lipid nanoparticle; AAV	Transient in function; No need to integrate into the genome	Moderate	Moderate	Low RNA stability; delivery component individually	Zetsche et al., 2015; Yin et al., 2016; Guimaraes et al., 2019; Liu et al., 2019a
Protein	Microinjection; electroporation; biolistic; microfluidic techniques; lipid nanoparticles	Fast; lower off-targeting	High	Low	Non-specific	Chen et al., 2016; Seki and Rutz, 2018; Suzuki et al., 2021

## Conclusion and Future Perspectives

Genome editing has entered a blooming period of development in recent years due to its extensive and effective application promise for scientific researches and disease treatment. The CRISPR/Cas9 system has been successfully applied to modify the genomes of different animals including mouse (Komor et al., 2016; Serneels et al., 2020; Takalo et al., 2020), rat (Serneels et al., 2020), monkey (Niu et al., 2014; Chen et al., 2015), cell lines including HT22 (Wang et al., 2019), N2a (Sun et al., 2017), stem cells (Paquet et al., 2016). More recently, there are already many publications on AD-related research mediated by CRISPR/Cas9, which mainly involves the use of this technology to construct AD model, screen pathogenic genes and treat AD *via* specific target genes (such as APP, BACE1, APOE4, CD33, GMF, and CysLT1R). However, considering potential off-target mismatches and specific tissue targeting in this technology, there are still numerous challenges to the eventual application of CRISPR-Cas9 to the clinical treatment of AD.

At present, a large number of researches are mainly focused on finding more efficient delivery systems. Protein-based CRISPR/Cas9 methods received more and more attention due to its low off-target effects, and higher editing efficiency. The development of CRISPR/Cas ribonucleoprotein (RNP) technology based on lipid nanoparticles (LNP) (Suzuki et al., 2021) has laid the foundation for the application of CRISPR/Cas9 technology in AD research and further clinical treatment. Notwithstanding these achievements, the numerous problems still exist. For instance, the AD animal models currently constructed are mostly rodents, however, the rodent model is not able to effectively and truly mimic the situation in the human brain, especially the occurrence of AD is closely related to the age factor since rodents generally have a short lifespan. Therefore, the application of results from animal models to the treatment of AD requires great care. Although the use of primates to build AD models by CRISPR/Cas9 strategy is likely to be a good strategy for simulating the age-dependent characteristics of AD, the long reproductive cycle of primates and the strict requirements for the growth environment also limit its application.

In summary, CRISPR/Cas9 is a masterpiece in gene editing technology this century. The gene scissors open new avenues in clinical gene therapy. Compared to other gene editing technologies, it has the advantages of short cycle, low cytotoxicity, low price, simple delivery, etc. Hence, all these characteristics make CRISPR-Cas9 system endowed with a broader application prospect in the clinical therapy of AD albeit taking into consideration of some drawbacks.

## Author Contributions

LL and XY wrote the manuscript with support from YC, MS, and HY. All authors contributed to the article and approved the submitted version.

## Conflict of Interest

The authors declare that the research was conducted in the absence of any commercial or financial relationships that could be construed as a potential conflict of interest.

## Publisher’s Note

All claims expressed in this article are solely those of the authors and do not necessarily represent those of their affiliated organizations, or those of the publisher, the editors and the reviewers. Any product that may be evaluated in this article, or claim that may be made by its manufacturer, is not guaranteed or endorsed by the publisher.
